# Movement behaviors during COVID-19 among Latin American/Latino toddlers and pre-schoolers in Chile, Mexico and the US

**DOI:** 10.1038/s41598-022-23850-1

**Published:** 2022-11-09

**Authors:** Alejandra Jáuregui, Deborah Salvo, Nicolas Aguilar-Farias, Anthony Okely

**Affiliations:** 1 Department of Physical Activity and Healthy Lifestyles, Center for Nutrition and Health Research, Instituto Nacional de Salud Pública, Cuernavaca, México; 2 Department of Kinesiology and Health Education, College of Education, The University of Texas at Austin, Austin, TX USA; 3 Prevention Research Center, Brown School, Washington University in St. Louis, St. Louis, MO USA; 4 Department of Physical Education, Sports and Recreation, Universidad de La Frontera, Temuco, Chile; 5 UFRO Activate Research Group, Universidad de La Frontera, Temuco, Chile; 6 Early Start and School of Health and Society, University of Wollongong, Wollongong, Australia; 7 Illawarra Health & Medical Research Institute, Wollongong, Australia

**Keywords:** Epidemiology, Paediatric research

## Abstract

Movement behaviors (physical activity, sedentary behavior, and sleep) have been impacted by the COVID-19 pandemic. We report changes in and factors that influenced movement behaviors during COVID-19 among Latin American/Latino children aged 1 to 5 years in Chile, Mexico, and the USA. We conducted a cross-sectional study between April and August 2020. Caregivers of 4,136 children (mean age [SD], 3.1 [1.4] years; 51% boys) reported family and household characteristics and changes in their child’s movement behaviors. The proportion of children who met the WHO Guidelines decreased significantly in all countries, with the largest declines in meeting the physical activity and screen time guidelines. Factors associated with negative changes in movement behaviors were being an older child, unable to attend an early childhood education and care service, higher parental education levels, not having the opportunity to play with someone, and not having access to spaces to play. The findings highlight the need to minimize disparities faced by families by providing access to early childhood education and care and safe places for children to play.

## Introduction

The COVID-19 pandemic has changed the daily lives of children, families, and communities^1,2^. Latin America and the USA have been epicenters for the pandemic^3^, which is due to widespread poverty and pre-existing socioeconomic inequalities in the region^4–7^. Despite comprising only 18% of the US population, Latino populations have comprised 30% of COVID-19 cases^8,9^. Similarly, around 30% of the total deaths globally due to COVID-19 have occurred in Latin America^10^. Consequently, Latin Americans/Latinos have faced a disproportionate burden that is likely to last well beyond the post-vaccination period.

In 2019 the World Health Organization (WHO) released guidelines for physical activity (PA), sedentary behavior, and sleep for children under five^11^. These guidelines acknowledge the interrelationships between these movement behaviors and reinforce that “the whole day matters” when promoting healthy levels of these behaviors. Subsequent research has confirmed that although meeting the recommended levels of these behaviors was associated with better health, only 10% of toddlers and 15% of preschoolers met the combined guidelines for these behaviors^12^.

Studies have documented the impact of the pandemic on movement behaviors in adults^13^ and school-age children and youth^14^ in North America, but there has been little empirical evidence of the impact on children under the age of 5, especially among Latin America/Latino populations^15^. Previous studies have shown that physical activity is associated with better psychosocial health and social skills in preschoolers^16,17^. More screen time has been associated with greater emotional and behavioral problems^18^, while shorter sleep has been related to higher levels of adiposity and poorer emotional regulation. ^19,20^ The closure of early childhood education and care services (ECEC), public spaces, parks, and playgrounds, combined with more parents/carers being unemployed or working from home, has likely impacted the movement behaviors, and in turn the health and wellbeing, of young children. A global study has shown that the impact of the COVID-19 pandemic affected differently children depending on their country of origin^15^. Therefore, understanding this impact and the contributing factors will guide the promotion of healthier levels of movement behaviors among children under 5 years. This study examined the changes in movement behaviors (PA, sedentary behavior, and sleep) during COVID-19 among Latin American/Latino children aged 1 to 5 years in Chile, Mexico, and the USA. Child, caregiver, and household factors associated with movement behaviors during COVID-19 were also examined.

## Methods

An online survey was conducted with the main caregivers of 1- to 5-year-old children in Chile, Mexico, and the USA. The study was promoted through social media (Facebook, Twitter, Instagram), text messages (i.e., WhatsApp and SMS), and emails. Potential participants accessed a personalized link for each study site to obtain details about the study and provided their informed consent to participate.

The inclusion criteria were: 1) living in Chile, Mexico, or the USA, 2) being the main caregiver of a 1- to 5-year-old child, 3) living with the child most of the time before and during the COVID-19 pandemic, and 4) only in the USA, identifying as Latino/Hispanic (defined as being of Latin American origin or descend).

Data were collected and managed in the three sites using REDCap (Research Electronic Data Capture)^2^.

Data collection occurred during the early stages of the pandemic, soon after the closure of early childhood education and care (ECEC) centers (Chile: March 30th to April 27th, 2020; Mexico: April 30th to July 27th, 2020; USA: May 14th to August 30th, 2020). ECEC centers were still closed in the three sites at the end of the data collection period.

### Sociodemographic variables

The following sociodemographic variables, which have been found to be associated with movement behaviors, were assessed: child sex, age, enrolment in ECEC (yes/no), whether the child usually played with someone or alone, access to electronic devices (none, 1 to 2, 3 or more), electronic devices in the room where the child usually sleeps (yes/no), and parental restrictions in the use of electronic devices (yes/no). When the adult was the caregiver of more than one child aged 1–5 years, the caregiver was asked to answer for only one child. Caregiver characteristics included age, sex, and educational level. Household characteristics included housing type (house, condominium or apartment, or other), number of adults and number of children ≤ 18 years at home, available space to play (yes/no), available backyard (yes/no), squared meters per person at home, and income level. In Chile, households were classified into low (< 530 USD), medium (≥ 530- < 1830 USD), and high (≥ 1830 USD) income. In the USA, households with an income < 200% of the 2019 federal poverty level were classified as low, those between 200 and 399% were classified as medium, and those > 400% were classified as high. In Mexico, households were categorized as high, medium, or low income using a validated questionnaire^21^. The area of residence (urban/rural) was also asked. Finally, caregivers reported whether they were in home confinement and the number of days they allowed their child to use electronic devices for education or to entertain or calm their child before and during COVID-19.

### Movement behaviors

Caregivers were asked to report their child’s time (in hours and minutes per day) spent in PA (total PA and moderate- to vigorous-intensity PA [operationalized as energetic play]), sedentary behaviors (sitting time and screen time [ST]), and sleep in a typical week before and during COVID-19. Questions were based on the WHO recommendations for each behavior^11^ and had been tested and refined in 22 countries as part of the pilot phases of the SUNRISE Study^22–25^. We calculated the time (min/d) spent in each behavior before and during COVID-19 and the difference between these two periods. We also estimated the proportion of children meeting WHO movement behavior guidelines for children under 5 at both periods^11^.

During both time periods, sleep quality was assessed with the question “How would you rate your child’s sleep quality?”, with Likert scale response options between 1 and 7 and a higher score indicating better sleep quality. Children with a sleep quality ≥ 4 were considered as having good sleep quality. We calculated the proportion of children with good sleep quality in both periods and the difference between these periods.

### Statistical analysis

Means and standard deviations, and frequencies and percentages were used to describe the samples. For each country, the total time in movement behaviors and the proportion of children who met the WHO movement behavior guidelines before and during COVID-19 were compared using t-tests and proportion tests, respectively. Multiple linear regressions were used to assess the association between each sociodemographic variable and changes in movement behaviors for each country. All analyses were adjusted for the other sociodemographic variables. Logistic regressions were used to explore the association between factors and changes in movement behavior compliance (supplementary file). All data preparation and analyses were conducted with Stata 15.0 (College Station, TX: StataCorp LLC). The level of statistical significance was *p* < 0.05.

### Ethical approval

The study was approved by the Scientific Ethics Committee for each study site independently (Universidad de La Frontera, Chile [ORD.: 009-2020], the National Institute of Public Health of Mexico [CI: 1661], and Washington University in St. Louis [IRB ID:202005074]), in accordance with the Declaration of Helsinki.

## Results

In total, 4136 children were included in the final analyses, 3045 in Chile, 632 in Mexico, and 459 in the USA. Around half the sample was female, and the average child age was 3.1 (1.4) years. A larger proportion of caregivers in Chile were younger (44.4% under 30 years of age). In Mexico, a larger proportion of caregivers had a university degree (75.8%), households were bigger (46.0%, ≥ 25 squared meters per person), and from a high-income level (46.3%). In the USA, fewer Latino children were enrolled in ECEC (12.4% for 1- to 2-year-olds and 48.7% for 3- to 5-year-olds), more lived in apartments (35.2%), were from lower-income (80.5%) and were located in rural areas (26.8%) (see Table 1).

**Table 1:** Sample characteristics across the three countries.

	Chile (N = 3045)	Mexico (N = 632)	US Latinos (N = 459)
**Child characteristics**			
Sex, n (%)			
Female	1498 (49.2)	297 (47.0)	236 (51.4)
Male	1547 (50.8)	335 (53.0)	223 (48.6)
Child’s age, n (%)			
1–2 y	1198 (39.3)	215 (34.0)	145 (31.6)
3–4 y	1302 (42.8)	257 (40.7)	218 (47.5)
5 y	545 (17.9)	160 (25.3)	96 (20.9)
Children enrolled in early childcare centers, n (%)			
1–2 y	699 (56.2)	117 (50.9)	26 (12.4)
3–5 y	1786 (93.3)	386 (83.2)	200 (48.7)
Children usually plays with someone, n (%)			
Yes	2295 (75.4)	470 (74.4)	343 (74.7)
No	750 (24.6)	162 (25.6)	116 (25.3)
Access to electronic devices, n (%)			
None	33 (1.1)	13 (2.1)	30 (6.5)
1 to 2	2145 (70.4)	391 (61.9)	351 (76.5)
3 or more	867 (28.5)	228 (36.1)	78 (17.0)
Electronic device in child’s room -Yes, n (%)	1598 (52.5)	289 (45.7)	197 (42.9)
Restrictions in the use of electronic devices, n (%)	1990 (65.4)	457 (72.3)	334 (72.8)
**Caregiver characteristics**			
Age, n (%)			
30 years or less	1353 (44.4)	199 (31.5)	140 (30.5)
31–40 years	1486 (48.8)	371 (58.7)	275 (59.9)
41 years or more	206 (6.8)	62 (9.8)	44 (9.6)
Sex, n (%)			
Female	2940 (96.6)	584 (92.4)	448 (97.6)
Male	105 (3.4)	48 (7.6)	11 (2.4)
Education level, n (%)			
Incomplete high school or less	507 (16.7)	21 (3.3)	111 (24.2)
Complete high school or technical degree	2162 (71.0)	132 (20.9)	192 (41.8)
University degree	376 (12.4)	479 (75.8)	156 (34.0)
**Household characteristics**			
Housing type, n (%)			
House	2483 (81.5)	479 (75.8)	256 (55.8)
Condominium or Apartment	482 (15.8)	148 (23.4)	162 (35.3)
Other	80 (2.6)	5 (0.8)	41 (8.9)
Number of adults per home, mean (SD)	2.4 (1.1)	2.4 (1.1)	2.2 (0.9)
Number of children 18 years or younger per home, mean (SD)	1.7 (0.8)	1.6 (0.8)	2.3 (1.1)
Available space to play, n (%)	2971 (97.6)	601 (95.1)	414 (90.2)
Backyard, n (%)	2793 (91.7)	517 (81.8)	354 (77.1)
Squared meters per person at home, n (%)^a^			
< 11.7	974 (30.9)	161 (23.1)	250 (60.1)
≥ 11.7 to < 18.3	698 (22.1)	106 (15.2)	51 (12.3)
≥ 18.3 to < 25	603 (19.1)	109 (15.7)	52 (12.5)
≥ 25	882 (27.9)	320 (46.0)	63 (15.1)
Income level, n (%)			
Low	975 (32.0)	14 (2.2)	369 (80.4)
Medium	1493 (49.0)	325 (51.6)	78 (17.0)
High	577 (19.0)	292 (46.2)	12 (2.6)
Residence area, n (%)			
Urban	1692 (88.4)	622 (98.4)	336 (73.2)
Rural	353 (11.6)	10 (1.6)	123 (26.8)
Home Confinement—Yes, n (%)	2330 (76.5)	535 (84.7)	381 (83.0)

^a^ Sample size in the US, n = 416.

All movement behaviors changed across the three countries during the early stages of the COVID-19 pandemic (Table 2). Total PA decreased by about 20%, ST almost doubled, and sleep quality decreased by around 15 percentage points. Sleeping duration decreased in Chile by around 5 min per night. During COVID-19, about a third of children met the PA recommendations and less than 10% met the ST recommendations. No changes were observed in the proportion who met the sleep duration recommendations.

**Table 2:** Movement behaviors before and during COVID-19 in Chilean, Mexican, and US Latino children (1–5 y).

	Chile			Mexico			US Latinos		
	Before	During	*p* value^a^	Before	During	*p* value^a^	Before	During	*p* value^a^
**Physical Activity**									
Minutes per day, mean (SD)	214.7 (118.7)	169.4 (128.7)	** < 0.001**	230.9 (144.0)	174.3 (147.6)	** < 0.001**	203.8 (144.0)	164.3 (152.5)	** < 0.001**
Meeting recommendations, n (%)	1734 (57.0)	1141 (37.5)	** < 0.001**	382 (60.4)	231 (36.6)	** < 0.001**	208 (45.3)	156 (34.0)	** < 0.001**
**Screen time**									
Minutes per day, mean (SD)	99.1 (68.9)	182.9 (115.4)	** < 0.001**	98.2 (74.8)	190.0 (115.3)	** < 0.001**	107.5 (101.0)	196.0 (166.7)	** < 0.001**
Meeting recommendations, n (%)	401 (13.2)	129 (4.2)	** < 0.001**	99 (15.7)	23 (3.6)	** < 0.001**	79 (17.2)	38 (8.3)	** < 0.001**
**Sleep time**									
Hours per day, mean (SD)	10.9 (1.8)	11.0 (1.9)	**0.003**	10.3 (2.7)	10.1 (2.7)	0.156	9.1 (3.7)	9.2 (3.9)	0.252
Meeting time recommendations, n (%)	2150 (70.6)	2187 (71.8)	0.295	402 (63.6)	396 (62.7)	0.727	245 (53.4)	244 (53.2)	0.947
**Sleep quality**^**b**^									
Mean sleep quality, mean (SD)	5.7 (1.5)	4.9 (1.8)	** < 0.001**	5.9 (1.4)	4.9 (1.8)	** < 0.001**	5.5 (1.9)	4.6 (2.1)	** < 0.001**
Good sleep quality, n (%)	2706 (88.9)	2359 (77.5)	** < 0.001**	578 (91.5)	478 (75.6)	** < 0.001**	382 (83.2)	312 (68.0)	** < 0.001**
**All recommendations** , n (%)	145 (4.8)	32 (1.6)	** < 0.001**	36 (5.7)	10 (1.6)	** < 0.001**	15 (3.3)	2 (0.4)	** < 0.001**

^a^ *p* values indicate significant differences between the two measurement times (before and during the COVID-19 pandemic). Estimated using paired t-tests (for continuous variables) or proportion tests (for proportions).

^b^ Measured with a Likert scale where “1” indicates difficulty falling asleep, waking up several times throughout the night for long period, and being restless (coughing and turning, moving and kicking the bedclothes off), whereas “7” indicates falling asleep easily and within few minutes, sleeping well through the night, breathing normally and sleeping deeply. Those answering 4–7 were classified as with “good sleep”.

During COVID-19, more than half of the caregivers permitted more frequent use of electronic devices as an education means (Chile = 51.5%, Mexico = 68.8%, and USA = 53.8%) compared with before this period. About 60% (Chile = 56.5%, Mexico = 66.3%, and USA = 59.9%) and around 40% of caregivers (Chile = 38.4%, Mexico = 45.9%, and USA = 51.6%) used the devices more frequently to entertain or calm their child, respectively (data not shown) during this period compared to before COVID-19.

### Factors associated with changes in physical activity

In all countries, decreases in PA were consistently associated with reductions in sleep quality, inability to play with someone else, and not having an available space to play (Table 3). In Mexico and Chile, decreases in PA were also associated with increases in ST, being older, living in an apartment or condominium, and not having an available backyard. In Chile and USA Latinos, decreases in PA were associated with having higher educated caregivers. Regarding country-specific results, in Chile, decreases in PA were associated with being female, being enrolled in an ECEC, having older caregivers, living with fewer children, and living in urban areas. In Mexico, reductions in PA were more likely among children who lived in non-traditional residential structures (e.g., huts, motor homes, ranches) and among those who lived with fewer adults at home.

**Table 3:** Factors associated with changes in physical activity and screen time during COVID-19 across the three countries.

	Chile (n = 3045)		Mexico (n = 632)		US Latinos (n = 459)	
Associated factors	Physical activity (min/day)	Screen time (min/day)	Physical activity (min/day)	Screen time (min/day)	Physical activity (min/day)	Screen time (min/day)
	β (95% CI) ^a^	β (95% CI) ^b^	β (95% CI) ^a^	β (95% CI) ^b^	β (95% CI) ^a^	β (95% CI) ^b^
**Changes in other movement behaviors**						
Physical activity (min/day)	na	−0.2*** [−0.2,−0.2]	na	−0.2*** [−0.2,−0.1]	na	0.0 [−0.1,0.2]
Screen time (min/day)	−0.3*** [−0.3,−0.2]	na	−0.3*** [−0.4,−0.2]	na	0.0 [−0.1,0.2]	na
Sleep time (min/day)	−0.0 [−2.3,2.3]	−1.0 [−3.3,1.2]	−0.023 [−0.1,0.1]	−0.1 [−0.1,0.0]	0.0 [−0.1,0.1]	−0.0 [−0.2,0.1]
Sleep quality (1 point, 1 to 7 range)	2.0* [0.0,4.0]	−5.6*** [−7.5,−3.7]	9.9*** [4.5,15.3]	−4.1 [−8.9,0.8]	5.6 [−0.9,12.0]	−11.2*** [−17.3,−5.0]
**Child characteristics**						
Male sex, (Ref: Female)	3.3 [−3.1,9.7]	5.4 [−0.6,11.3]	7.3 [−9.2,23.9]	14.6* [1.8,27.4]	14.5 [−7.5,36.5]	1.5 [−21.8,24.7]
Age, (Ref: 1–2 y)						
3–4 y	−12.0** [−20.0,−3.9]	15.7*** [8.4,23.0]	−19.2 [−40.6,2.2]	27.8** [10.6,45.0]	−2.7 [−32.4,26.9]	5.7 [−24.3,35.8]
5 y	−18.4*** [−28.7,−8.0]	25.7*** [15.8,35.7]	−15.6 [−39.8,8.5]	19.0* [0.3,37.6]	−11.6 [−51.2,28.1]	19.0* [0.3,37.6]
Enrolled in early childcare center, (Ref: No)	−9.7* [−18.2,−1.3]	29.6*** [22.3,36.8]	−2.4 [−22.4,17.7]	25.6** [8.3,43.0]	−5.8 [−36.9,25.4]	−7.6 [−33.1,17.9]
Children usually plays with someone, (Ref: No)	9.6* [1.7,17.5]	−26.3*** [−34.2,−18.5]	15.9 [−2.7,34.4]	−43.2*** [−62.3,−24.2]	30.3* [3.0,57.5]	−7.6 [−37.7,22.6]
**Caregiver characteristics**						
Age, (Ref: < 30 years)						
31–40 years	−13.7*** [−21.1,−6.2]	5.5 [−1.3,12.4]	4.1 [−14.8,23.0]	−1.7 [−22.6,19.3]	20.4 [−6.9,47.7]	19.8 [−7.3,47.0]
41 years or more	−16.2** [−28.5,−4.0]	−9.5 [−21.4,2.4]	5.0 [−42.0,32.0]	−13.3 [−43.2,16.5]	−0.6 [−38.4,37.2]	− 14.4 [−22.9,51.6]
Male sex, (Ref: Female)	20.4* [3.3,37.5]	−6.5 [−20.1,7.1]	1.0 [−31.5,33.4]	−1.8 [−24.8,21.1]	−6.1 [−43.8,31.4]	17.7 [−38.1,73.6]
Education level, (Ref: Incomplete high school or less)						
Complete high school or technical degree	−8.2 [−18.3,1.8]	11.9** [3.3,20.5]	−0.4 [−38.4,37.6]	−4.7 [−46.8,37.4]	−33.0* [−63.4,−2.5]	−13.8 [−52.3,24.8]
University degree	−14.8* [−28.3,−1.4]	16.8** [4.4,29.3]	−2.5 [−37.5,32.5]	1.1 [−43.0,45.1]	−32.0* [−62.0,−2.0]	−5.6 [−46.1,34.9]
**Household characteristics**						
Housing type, (Ref: House)						
Apartment or Condominium	−20.5*** [−29.9,−11.1]	5.4 [−4.0,14.7]	−19.9* [−39.7,−0.1]	6.8 [−11.8,25.3]	3.5 [−24.4,31.5]	11.0 [−20.7,42.7]
Other	12.2 [−12.2,36.6]	−17.5* [−31.8,−3.2]	−95.6*** [−144.0,−47.2]	1.4 [−55.5,58.3]	41.8 [−12.6,96.1]	13.2 [−47.5,73.9]
Number of adults per home, (n)	−0.4 [−3.6,2.8]	−3.0 [−6.2,0.2]	11.8** [4.0,19.6]	−5.6 [−15.8,4.5]	−7.4 [−20.2,5.4]	3.5 [−9.8,16.8]
Number of children < 18y in the household, (n)	4.3* [0.0,8.5]	3.5 [−0.3,7.3]	−9.7 [−21.4,2.1]	−4.7 [−14.6,5.1]	−0.6 [−14.3,13.2]	−10.4 [−24.0,3.2]
Access to electronic devices, (Ref: None)						
1 to 2	na	51.7*** [35.8,67.6]	na	80.2*** [33.3,127.2]	na	38.4 [−3.7,80.4]
3 or more	na	60.8*** [44.0,77.7]	na	91.3*** [42.8,139.8]	na	80.6** [31.8,129.5]
Electronic device in the room, (Ref: No)	na	4.2 [−2.0,10.3]	na	14.4 [−2.4,31.2]	na	−0.5 [−27.8,26.8]
Limits in the use of electronic devices, (Ref: No)	na	−39.1*** [−45.8,−32.5]	na	−31.8*** [−48.2,−15.3]	na	−30.3* [−59.5,−1.2]
Available space to play, (Ref: No)	27.4*** [13.3,41.5]	−14.0 [−28.3,0.3]	11.7 [−21.4,44.8]	−27.4 [−61.0,6.3]	40.3* [8.0,72.7]	−48.0* [−89.6,−6.3]
Available backyard, (Ref: No)	3.5 [−9.5,16.6]	−3.8 [−16.8,9.2]	−5.9 [−29.3,17.5]	−20.8 [−43.1,1.5]	5.9 [−25.3,37.0]	32.6 [−3.5,68.8]
Income level, (Ref: Low)						
Medium	1.9 [−6.2,10.0]	0.4 [−6.9,7.8]	28.7 [−20.3,77.7]	36.4 [−75.4,148.3]	9.9 [−21.6,41.3]	−15.8 [−43.8,12.2]
High	5.1 [−5.3,15.5]	1.5 [−8.0,10.9]	41.3 [−10.8,93.4]	45.9 [−70.7,162.5]	−9.4 [−50.0,31.1]	17.8 [−26.8,62.4]
Rural area, (Ref: Urban)	40.3*** [29.8,50.7]	−7.6 [−16.3,1.1]	26.7 [−32.3,85.6]	−3.1 [−78.9,72.6]	−4.1 [−27.2,19.0]	4.6 [−24.6,33.7]
Home confinement, (Ref: No)	−3.3 [−10.8,4.2]	1.8 [−5.5,9.1]	−7.5 [−33.0,17.9]	−6.0 [−25.9,13.9]	6.5 [−21.1,34.2]	4.9 [−40.3,50.2]

***p < 0.001, **p < 0.01, *p < 0.05.

^a^ Adjusted for the variables listed in the table plus physical activity before COVID−19 and region within the country.

^b^ Adjusted for the variables listed in the table plus screen time before COVID−19 and region within the country.

### Factors associated with changes in screen-time

Across countries, increases in ST were associated with reductions in sleep quality, being older, having greater access to electronic devices, not having any parental-imposed restrictions in using such devices, and not having any available space to play (Table 3). In Chile and Mexico, increases in ST were also associated with reductions in PA, being older, being previously enrolled in ECEC, not being able to play with someone else, and living in an apartment or condominium. In Chile, high parental education and residing in non-traditional homes (e.g., huts, motor homes, ranches) were associated with increased ST during COVID-19.

### Factors associated with sleep duration and quality

In all countries, decreases in sleep duration were more likely among those who lived in apartments or condominiums (Table 4). In Mexico and Chile, decreases in sleep duration were also associated with reductions in sleep quality. In Chile, older children, those who were not previously enrolled in ECEC, and with more educated caregivers had higher decreases in sleep duration. Being a boy was associated with a greater decline in sleep duration in Mexico.

**Table 4:** Factors associated with changes in sleep duration and sleep quality during COVID−19 across the three countries.

	Chile (n = 3045)		Mexico (n = 632)		US Latinos (n = 459)	
Associated factors	Sleep duration (min/day)	Slee*p* quality (1 to 7 score)	Sleep duration (min/day)	Slee*p* quality (1 to 7 score)	Sleep duration (min/day)	Slee*p* quality (1 to 7 score)
	β (95% CI) ^a^	β (95% CI) ^b^	β (95% CI) ^a^	β (95% CI) ^b^	β (95% CI) ^a^	β (95% CI) ^b^
**Changes in other movement behaviors**						
Physical activity, (min/day)	0.0 [−0.0,0.1]	0.0 [−0.0,0.0]	0.0 [−0.0,0.1]	0.0* [0.0,0.0]	−0.0 [−0.1,0.1]	0.0* [0.0,0.0]
Screen time, (min/day)	−0.0 [−0.1,0.0]	−0.0*** [−0.0,−0.0]	0.0 [−0.1,0.1]	−0.0* [−0.0,−0.0]	0.0 [−0.2,0.2]	−0.0* [−0.0,−0.0]
Sleep duration, (min/day)	na	0.0*** [0.0,0.0]	na	0.0*** [0.0,0.0]	na	−0.0 [−0.0,0.0]
Sleep quality, (1 point, 1 to 7 range)	9.4*** [7.1,11.7]	na	14.5*** [9.4,19.6]	na	−1.2 [−8.2,5.8]	na
**Child characteristics**						
Male sex, (Ref: Female)	−1.5 [−7.6,4.7]	0.0 [−0.1,0.1]	−19.2* [−33.9,−4.5]	0.2 [−0.0,0.4]	−3.4 [−28.8,22.1]	−0.0 [−0.3,0.3]
**Age, (Ref: 1–2 y)**						
3–4 y	−16.0*** [−24.8,−7.1]	0.2* [0.0,0.3]	−15.8 [−34.4,2.8]	0.3 [−0.0,0.6]	−8.0 [−37.2,21.1]	0.2 [−0.2,0.6]
5 y	−16.1** [−27.2,−5.1]	0.1 [−0.0,0.3]	2.4 [−19.7,24.6]	−0.1 [−0.4,0.3]	−15.3 [−53.5,22.9]	0.2 [−0.3,0.8]
Enrolled in early childcare center, (Ref: No)	10.4* [1.7, 19.1]	−0.0 [−0.2,0.1]	4.0 [−15.2,23.2]	−0.2 [−0.6,0.1]	20.6 [−9.2,50.4]	−0.3 [−0.7,0.1]
Children usually plays with someone, (Ref: No)	2.9 [−5.0, 10.8]	−0.0 [−0.1,0.1]	0.6 [−19.1,20.4]	−0.0 [−0.3,0.2]	−13.8 [−44.8,17.3]	0.3 [−0.1,0.7]
**Caregiver characteristics**						
Age, (Ref: < 30 years)						
31–40 years	−0.6 [−7.8, 6.5]	0.1 [−0.0,0.2]	4.0 [−16.3,24.2]	−0.0 [−0.3,0.3]	−1.8 [−28.6,25.1]	−0.1 [−0.4,0.3]
41 years or more	−10.1 [−24.5, 4.3]	0.2 [−0.0,0.4]	−14.7 [−41.4,12.0]	−0.2 [−0.6,0.3]	−19.9 [−63.7,24.0]	−0.0 [−0.7,0.7]
Male sex, (Ref: Female)	−3.6 [−18.2, 11.0]	0.3* [0.0,0.6]	19.3 [−8.7,47.4]	0.1 [−0.3,0.5]	−0.9 [−61.0,59.2]	0.9 [−0.5,2.3]
Education level, (Ref: Incomplete high school or less)						
Complete high school or technical degree	−11.8* [−22.4,−1.1]	0.1 [−0.1,0.3]	−15.0 [−58.9,28.9]	−0.0 [−0.7,0.7]	16.9 [−20.3,54.1]	−0.2 [−0.6,0.3]
University degree	−21.1** [−34.4,−7.8]	0.1 [−0.1,0.4]	−4.7 [−46.0,36.5]	0.3 [−0.4,0.9]	12.4 [−29.2,54.0]	0.1 [−0.5,0.6]
Household characteristics						
Housing type, (Ref: House)						
Apartment or Condominium	−4.0 [−13.3,5.3]	−0.0 [−0.2,0.2]	−9.3 [−29.3,10.7]	0.3 [−0.0,0.6]	−31.3* [−62.5,−0.2]	0.2 [−0.2,0.6]
Other	4.0 [−19.3, 27.4]	−0.0 [−0.4,0.3]	−0.5 [−80.5,79.5]	−0.0 [−1.5,1.4]	−5.5 [−47.3,36.3]	0.2 [−0.4,0.8]
Number of adults per home, mean (SD)	1.3 [−2.1,4.7]	−0.0 [−0.1,0.0]	−3.3 [−12.2,5.7]	0.0 [−0.1,0.2]	0.4 [−17.3,18.2]	0.0 [−0.2,0.2]
Number of children < 18y in the household, mean (SD)	0.6 [−3.6, 4.7]	−0.0 [−0.1,0.0]	−1.7 [−11.7,8.2]	0.0 [−0.1,0.2]	0.4 [−12.2,13.0]	−0.2 [−0.3,0.0]
Access to electronic devices, (Ref: None)						
1 to 2	−5.1 [−29.5,19.2]	0.1 [−0.4,0.6]	−32.0 [−78.3,14.3]	0.6 [−0.3,1.5]	−11.9 [−61.0,37.1]	−0.2 [−0.8,0.4]
3 or more	−5.2 [−30.3,19.8]	0.1 [−0.4,0.6]	−38.7 [−87.8,10.4]	0.5 [−0.5,1.4]	−45.4 [−99.8,8.9]	−0.2 [−1.0,0.5]
Electronic device in the room, (Ref: No)	−1.9 [−8.4,4.5]	−0.1 [−0.2,0.0]	6.9 [−8.2,22.0]	−0.2 [−0.4,0.1]	0.2 [−24.3,24.7]	−0.4* [−0.8,−0.0]
Limits in the use of electronic devices, (Ref: No)	−0.8 [−7.3,5.8]	−0.1 [−0.2,0.1]	−3.0 [−20.4,14.6]	0.1 [−0.1,0.4]	8.9 [−19.0,36.8]	0.1 [−0.5,0.3]
Available space to play, (Ref: No)	−13.3 [−28.1, 1.5]	0.6*** [0.3,0.8]	−30.5 [−65.7,4.6]	0.7** [0.1,1.2]	−20.1 [−57.2,17.1]	0.1 [−0.4,0.7]
Available backyard, (Ref: No)	9.1 [−4.2, 22.4]	−0.1 [−0.3,0.1]	6.9 [−17.9,31.6]	−0.2 [−0.6,0.2]	−7.2 [−44.0,29.6]	0.2 [−0.3,0.6]
Income level, (Ref: Low)						
Medium	3.6 [−4.6, 11.7]	0.1 [−0.1,0.2]	−46.9 [−125.7,31.9]	0.2 [−0.8,1.2]	−3.5 [−31.9,39.0]	0.0 [−0.5,0.5]
High	3.7 [−6.0, 13.3]	0.3** [0.1,0.5]	−42.9 [−123.8,38.0]	0.4 [−0.7,1.5]	−3.9 [−69.6,61.7]	0.6 [−0.4,1.6]
Rural area, (Ref: Urban)	5.1 [−4.5, 14.7]	0.1 [−0.1,0.2]	5.8 [−58.0,69.6]	0.2 [−0.7,1.0]	3.5 [−25.5,32.4]	−0.0 [−0.4,0.4]
Home confinement, (Ref: No)	−4.1 [−11.3, 3.2]	−0.1 [−0.2,0.0]	3.1 [−19.0,25.2]	−0.0 [−0.4,0.3]	6.5 [−32.6,45.5]	−0.1 [−0.5,0.3]

***p < 0.001, **p < 0.01, *p < 0.05.

^a^ Adjusted for the variables listed in the table plus sleep duration before COVID-19 and region within the country.

^b^ Adjusted for the variables listed in the table plus sleep quality before COVID-19 and region within the country.

Increases in ST and decreases in PA were associated with decreases in sleep quality across all countries (Table 4). In Chile and Mexico, increases in sleep quality were also associated with increases in sleep time and with having available space to play at home. In Chile, male children and those from high SES families had better sleep quality. In the USA, having electronic devices in the child’s room was associated with worse sleep quality during the pandemic.

## Discussion

Our study showed clear changes in PA, ST, and sleep quality among preschool Latin American/Latino children from Chile, Mexico, and the USA during COVID-19^14,26,27^. Several child, caregiver, and household environment characteristics were consistently associated with unhealthy changes in all movement behaviors across countries during the pandemic. At the child level, these included being older and not being able to attend ECEC. Higher parental education levels, not having the opportunity to play with someone, and not having access to spaces to play were important caregiver and household environment factors that were negatively associated with movement behaviors among Latin American/Latino preschool children during the pandemic.

Some of our results are consistent with reports from other countries among school-aged children. This includes a decline in PA^14,26^ and an increase in ST during COVID-19^14,27^. This is likely due to children not being able to go outdoors, not attending their ECEC in-person, and often switching to online educational activities or no educational provision^28^, and having increased access to electronic media devices during COVID-19. It has been shown that children are more active and less sedentary the more time they spend outdoors^29–31^, and, in some countries, are more active at ECEC than at home^32^. Outdoor play is important in maintaining good sleep quality, which may be explained by the effect of sunlight exposure on sleep and circadian rhythms^33^. In our study, increases in ST and decreases in PA were greater among preschoolers than toddlers, which is likely due to preschoolers having greater changes in their daily routines as a result of the closure of ECEC services, given their higher attendance rates than toddlers. Parents working from home may also allow their children greater access to electronic media devices to keep them busy while the parent works. This could explain why higher parental education was associated with more negative changes in movement behaviors in our study, as higher educated adults were most likely to have jobs allowing them to work from home during COVID-19^34^.

We found strong and consistent relationships across the three countries for changes in PA, ST, and sleep quality, but not for sleep duration. As expected, PA increases were associated with positive changes in sleep quality and decreases in ST^35–37^. This reinforces that these behaviors are co-dependent and should be promoted as part of a healthy movement pattern over 24 h, as suggested by the WHO global guidelines^11^. The null association for sleep duration can be explained by the lack of change in sleep duration, unlike the difference in sleep quality, which was more profound and likely due to children going to bed and waking up later, a pattern of sleeping associated with poorer health outcomes^38^.

Although most of our findings are not unexpected, documenting the adverse effects of COVID-19 on Latin American/Latino preschool children has added value beyond the description of temporal changes in movement behavior patterns during COVID-19. The impact of COVID-19 on the health and wellbeing of children is expected to be long-lasting^39–41^. Before the pandemic, Latin American countries already faced a disproportionate double burden from non-communicable diseases^42,43^, which resulted from rapid demographic and health transitions (including several large waves of migration to the USA) ^44^, in an environment with pervasive social and economic inequalities^45,46^. Understanding if and how COVID-19 has contributed to widening health inequalities and identifying factors associated with worse or better outcomes can provide critical information to develop tailored programs for promoting resilience and healthy movement behaviors during and after the pandemic. Our results suggest that such strategies must employ a multilevel, equity-driven approach.

One key finding of our study was the role social and environmental opportunities play in facilitating healthy movement behaviors during the pandemic. In line with other studies^29,30^, opportunities for the child to play with other children or adults is an important factor associated with healthier levels of all three movement behaviors. Similarly, having a space for children to play was associated with more favorable changes in movement behaviors. It is possible that low-income families or those living in highly populated urban areas may be less likely to have designated “play spaces” in their private residences for their children to stay active and to limit screen-time and prolonged sitting. Usually, the lack of private space for play can be addressed by using public open recreation spaces such as parks, playgrounds or public squares. However, during COVID-19, many local governments either limited or closed access to these public facilities—a situation which has likely resulted in a greater gap between high- and low-income families in access to health-promoting spaces. Furthermore, the social isolation that results from lockdowns or stay-at-home orders may more significantly impact Latin American/Latino children and families given their strong collectivistic (community-oriented) identity. In fact, there is evidence that for adults, use and access to places that facilitate social interaction is a key driver of PA behaviors^47,48^.

We found that the level of access to electronic devices was associated with ST across all countries. A challenge faced by many caregivers is the ubiquity of screen devices. The mean number of connected devices owned by a USA household was 10.4 in 2020^49^. This provides children with many opportunities for ST, making it more challenging for caregivers to monitor the amount of ST for their child. It was encouraging that around two-thirds of caregivers set limits on the amount of time their child could use electronic devices and that they felt that setting limits effectively reduced their child’s ST. This strategy has been shown to be highly effective in lowering ST among children^50,51^ with the additional benefits of greater social interactions^52^, which is important in language and social development^53,54^.

The results of this study should be interpreted with consideration of the limitations. Although we asked caregivers to retrospectively report on their children’s patterns of movement behaviors before and during the pandemic, the cross-sectional survey design precludes us from inferring causality. Because our measures are based on self-report, and caregivers reported on behalf of their children, there may be some degree of information bias. Further, we recruited our sample using online social media. Although the income and education distribution of participants in our sample broadly resembled those of the general population in Chile and among USA Latinos, there is potential for selection bias as respondents are more likely to be those with access to and frequent use of online media.

Our study had several strengths. We used standardized methods to collect comparable data across the three countries. This is the first study documenting changes in movement behaviors during COVID-19 among preschool children of Latin American origin or descent. We achieved robust sample sizes across all sites. Our results showed consistent findings across countries, suggesting broad applicability across Latin America, with potential for developing cross-national recommendations. At the same time, the country-specific results provide important information for developing locally tailored plans and actions to promote healthy living opportunities during and after a major global crisis like COVID-19.

## Conclusions

In this study of Latin American and Latino children during the first wave of the COVID-19 pandemic, there was a decline in the proportion of children who met the WHO global guidelines for PA and ST and sleep quality compared with pre-COVID-19. Parents with higher education levels and those with older children were more likely to report unhealthy movement behavior changes. Not having access to an ECEC or to a space to play and not having the opportunity to play with someone were negatively associated with movement behaviors during the pandemic. This research provides further evidence on the impact the closure of ECEC centers had on children’s physical and social health and the challenges parents faced with having their child at home during this pandemic stage, especially with managing a healthy level of screen time.

## Supplementary Information

Supplementary Information.

## Data Availability

The datasets used and/or analysed during the current study are available from the corresponding author on reasonable request.
