# Supplementary material for: Movement behaviors during COVID-19 among Latin American/Latino toddlers and pre-schoolers in Chile, Mexico and the US
Source: Sci Rep. 2022 Nov 9;12:19156. doi: 10.1038/s41598-022-23850-1 (PMC9646275; doi:10.1038/s41598-022-23850-1)
Supplement: Supplementary file 1 — Supplementary Information. [file 41598_2022_23850_MOESM1_ESM.docx]

**Supplementary files**

**Movement behaviors during COVID-19 among Latin American/Latino toddlers and pre-schoolers in Chile, Mexico and the US**

Alejandra Jáuregui, Sc.D,^1^ Deborah Salvo, PhD, ^1, 2^ Nicolas Aguilar Farias, PhD,^3,4^ Anthony Okely, EdD.^5,6^

^1^ Department of physical Activity and Healthy Lifestyles, Center for Nutrition and Health Research, Instituto Nacional de Salud Pública. Cuernavaca, México.

^2^ Prevention Research Center, Brown School, Washington University in St. Louis. St. Louis, MO, USA.

^3^ Department of physical education, sports and recreation. Universidad de La Frontera. Temuco, Chile.

^4^UFRO Activate Research Group, Universidad de La Frontera, Chile

^5^ Early Start and School of Health and Society, University of Wollongong, Australia

^6^ Illawarra Health & Medical Research Institute, Wollongong, Australia

**Supplementary eTable 1. Logistic regression of factors associated with meeting physical activity guidelines during COVID-19 across sample characteristics, by country.**

|  | **Chile**  **(n=3045)** | **Mexico**  **(n=632)** | **US Latinos**  **(n=459)** |
| --- | --- | --- | --- |
| **Associated factors** | **OR (95% CI) ^a^** | **OR (95% CI)^a^** | **OR (95% CI)^a^** |
| **Meeting other movement behavior recommendations** |  |  |  |
| Screen time | 0.9  [0.6,1.3] | 1.6  [0.6,4.3] | 0.5  [0.2,1.0] |
| Sleep time | 1.1  [0.9,1.3] | 1.4  [0.9,2.1] | 0.8  [0.5,1.3] |
| **Child characteristics** |  |  |  |
| Male sex, (Ref: Female) | 1.1  [0.9,1.3] | 1.2  [0.8,1.7] | 1.6  [1.0,2.5] |
| Age, (Ref: 1-2 y) |  |  |  |
| 3-4 y | 0.5***  [0.4,0.7] | 0.5**  [0.3,0.8] | 0.7  [0.4,1.2] |
| 5 y | 0.5***  [0.4,0.6] | 0.5**  [0.3,0.8] | 0.4*  [0.2,0.9] |
| Enrolled in early childcare centre | 0.8**  [0.6,0.9] | 0.8  [0.5,1.2] | 0.6*  [0.3,0.9] |
| Children usually plays with someone | 1.7***  [1.4,2.1] | 2.1**  [1.3,3.3] | 1.6  [0.9,3.0] |
| **Caregiver characteristics** |  |  |  |
| Age, (Ref: <30 years) |  |  |  |
| 31-40 years | 0.6***  [0.5,0.8] | 0.8  [0.5,1.2] | 1.7  [1.0,2.8] |
| 41 years or more | 0.6*  [0.5,0.8] | 0.9  [0.4,1.9] | 0.9  [0.4,2.2] |
| Male sex, (Ref: Female) | 1.6*  [1.0,2.6] | 1.2  [0.6,2.3] | 1.7  [0.6,5.0] |
| Education level, (Ref: Incomplete high school or less) |  |  |  |
| Complete high school or technical degree | 0.8  [0.6,1.1] | 2.2  [0.7,7.3] | 0.9  [0.5,1.7] |
| University degree | 0.8  [0.5,1.1] | 2.0  [0.6,6.1] | 0.9  [0.4,1.8] |
| **Household characteristics** |  |  |  |
| Housing type, (Ref: House) |  |  |  |
| Apartment or Condominium | 0.6***  [0.4,0.8] | 0.7  [0.4,1.2] | 0.7  [0.4,1.2] |
| Other | 1.7  [1.0,2.9] | 0.6  [0.1,3.4] | 1.5  [0.7,3.5] |
| Number of adults per home, mean (SD) | 1.0  [0.9,1.1] | 1.2  [1.0,1.4] | 0.8  [0.6,1.1] |
| Number of children <18y in the household, mean (SD) | 1.0  [0.9,1.1] | 0.8  [0.6,1.1] | 1.0  [0.8,1.3] |
| Available space to play | 1.7*  [1.2,2.6] | 2.4*  [1.1,5.1] | 2.1*  [1.0,4.5] |
| Available backyard | 1.3  [0.9,1.8] | 1.4  [0.8,2.5] | 0.9  [0.5,1.6] |
| Income level (Ref: Low) |  |  |  |
| Medium | 1.0  [0.8,1.2] | 0.4  [0.1,1.7] | 1.0  [0.5,2.1] |
| High | 1.0  [0.7,1.3] | 0.5  [0.1,1.9] | 1.8  [0.4,7.8] |
| Rural area, (Ref: Urban) | 2.3***  [1.7,3.0] | 0.9  [0.2,3.7] | 0.9  [0.5,1.6] |
| Confinement | 0.9  [0.7,1.1] | 1.0  [0.5,1.7] | 1.2  [0.6,2.1] |

OR, Odds Ratio; 95% CI, 95% Confidence Intervals

^a^Models adjusted for the variables listed in the table, meeting physical activity recommendation before COVID-19 and country region

**Supplementary eTable 2. Logistic regression of factors associated with meeting screen time guidelines during COVID-19 across sample characteristics, by country.**

|  | **Chile**  **(n=3045)** | **Mexico**  **(n=558)** | **US Latinos**  **(n=459)** |
| --- | --- | --- | --- |
| **Associated factors** | **OR (95% CI) ^a^** | **OR (95% CI)^a^** | **OR (95% CI)^a^** |
| **Meeting other movement behavior recommendations** |  |  |  |
| Physical activity | 1.0  [0.6,1.5] | 2.7  [0.7,10.6] | 0.2*  [0.1,0.8] |
| Sleep time | 0.5**  [0.4,0.8] | 1.8  [0.5,6.9] | 0.2*  [0.1,0.7] |
| **Child characteristics** |  |  |  |
| Male sex, (Ref: Female) | 0.9  [0.6,1.4] | 1.9  [0.6,6.8] | 0.6  [0.2,1.6] |
| Age, (Ref: 1-2 y) |  |  |  |
| 3-4 y | 0.8  [0.5,1.3] | 1.8  [0.4,7.5] | 0.6  [0.2,1.9] |
| 5 y | 0.5  [0.3,1.1] | 1.0  [0.1,5.6] | 1.3  [0.2,6.8] |
| Enrolled in early childcare centre | 0.6  [0.4,1.0] | 1.1  [0.2,5.3] | 0.7  [0.2,2.3] |
| Children usually plays with someone | 1.2  [0.4,1.0] | 1.2  [0.3,5.8] | 2.4  [0.7,8.8] |
| **Caregiver characteristics** |  |  |  |
| Age, (Ref: 30 years or less) |  |  |  |
| 31-40 years | 0.8  [0.5,1.3] | 0.2  [0.1,1.1] | 0.3  [0.1,1.0] |
| 41 years or more | 1.2  [0.5,2.9] | 0.3  [0.0,3.5] | 0.2  [0.0,1.4] |
| Male sex, (Ref: Female) | 1.3  [0.4,3.9] | 1.0  [1.0,1.0] | 1.0  [0.0,38.9] |
| Education level, (Ref: Incomplete high school or less) |  |  |  |
| Complete high school or technical degree | 0.5*  [0.3,0.9] | 0.1  [0.0,2.8] | 0.9  [0.3,3.2] |
| University degree | 0.5  [0.2,1.2] | 0.7  [0.0,13.7] | 0.7  [0.1,3.7] |
| **Household characteristics** |  |  |  |
| Housing type, (Ref: House) |  |  |  |
| Apartment or Condominium | 1.0  [0.5,2.0] | 0.1*  [0.0,0.9] | 1.2  [0.4,3.6] |
| Other | 1.3  [0.5,3.5] | 1.0  [1.0,1.0] | 0.5  [0.0,6.3] |
| Number of adults per home, mean (SD) | 1.1  [0.9,1.3] | 1.1  [0.6,2.1] | 0.7  [0.4,1.4] |
| Number of children <18y in the household, mean (SD) | 1.4**  [1.1,1.7] | 1.1  [0.5,2.4] | 0.9  [0.5,1.6] |
| Access to electronic devices, [Ref: None] |  |  |  |
| 1 to 2 | 0.1***  [0.0,0.4] | 0.1*  [0.0,0.6] | 0.2*  [0.1,0.8] |
| 3 or more | 0.1***  [0.0,0.3] | 0.1*  [0.0,0.8] | 0.0**  [0.0,0.5] |
| Electronic device in the room | 1.1  [0.7,1.6] | 0.2*  [0.0,0.9] | 0.7  [0.2,2.2] |
| Limits in the use of electronic devices | 1.7*  [1.1,2.9] | 0.5  [0.1,1.9] | 1.6  [0.4,6.1] |
| Available space to play | 1.8  [0.6,5.2] | 1.8  [0.2,18.4] | 2.0  [0.4,9.7] |
| Available backyard | 1.2  [0.5,2.7] | 0.6  [0.1,5.3] | 0.4  [0.1,1.7] |
| Income level (Ref: Low) |  |  |  |
| Medium | 1.0  [0.6,1.6] | 3.8  [0.9,15.5] | 1.3  [0.3,6.3] |
| High | 0.9  [0.5,2.0] | 1.0  [1.0,1.0] | 1.7  [0.5,5.8] |
| Rural area, (Ref: Urban) | 1.8*  [1.0,3.1] | 1.0  [1.0,1.0] | 1.7  [0.5,5.8] |
| Confinement | 1.0  [0.6,1.7] | 0.6  [0.1,3.6] | 0.5  [0.1,1.8] |

OR, Odds Ratio; 95% CI, 95% Confidence Intervals

^a^Models adjusted for the variables listed in the table, meeting screen time recommendation before COVID-19 and country region

**Supplementary eTable 3. Logistic regression of factors associated with meeting sleep duration guidelines during COVID-19 across sample characteristics, by country.**

|  | **Chile**  **(n=3045)** | **Mexico**  **(n=632)** | **US Latinos**  **(n=459)** |
| --- | --- | --- | --- |
| **Associated factors** | **OR (95% CI) ^a^** | **OR (95% CI)^a^** | **OR (95% CI)^a^** |
| **Meeting other movement behavior recommendations** |  |  |  |
| Physical activity | 1.1  [0.9,1.3] | 1.1  [0.8,1.7] | 0.8  [0.6,1.3] |
| Screen time | 0.8  [0.5,1.0] | 2.1  [0.7,6.7] | 0.5  [0.2,1.1] |
| **Child characteristics** |  |  |  |
| Male sex, (Ref: Female) | 0.9  [0.8,1.1] | 0.8  [0.5,1.1] | 1.2  [0.8,1.9] |
| Age, (Ref: 1-2 y) |  |  |  |
| 3-4 y | 1.7***  [1.4,2.1] | 1.2  [0.7,1.8] | 1.4  [0.8,2.2] |
| 5 y | 1.8***  [1.4,2.4] | 1.9*  [1.1,3.3] | 1.6  [0.8,3.3] |
| Enrolled in early childcare center | 0.9  [0.8,1.2] | 0.9  [0.6,1.3] | 1.0  [0.6,1.7] |
| Children usually plays with someone | 1.3**  [1.1,1.6] | 1.1  [0.7,1.7] | 1.1  [0.7,1.8] |
| **Caregiver characteristics** |  |  |  |
| Age, (Ref: 30 years or less) |  |  |  |
| 31-40 years | 1.0  [0.8,1.3] | 1.1  [0.7,1.6] | 1.3  [0.8,2.2] |
| 41 years or more | 0.8  [0.6,1.2] | 0.7  [0.4,1.5] | 1.0  [0.4,2.3] |
| Male sex, (Ref: Female) | 0.8  [0.5,1.2] | 1.0  [0.5,2.1] | 2.2  [0.6,8.0] |
| Education level, (Ref: Incomplete high school or less) |  |  |  |
| Complete high school or technical degree | 0.9  [0.7,1.1] | 0.9  [0.3,2.6] | 1.0  [0.6,1.8] |
| University degree | 0.7  [0.5,1.0] | 1.3  [0.4,3.5] | 1.7  [0.9,3.1] |
| **Household characteristics** |  |  |  |
| Housing type, (Ref: House) |  |  |  |
| Apartment or Condominium | 0.8  [0.6,1.1] | 1.2  [0.7,2.1] | 0.8  [0.5,1.3] |
| Other | 1.0  [0.6,1.7] | 1.5  [0.2,13.6] | 1.0  [0.5,2.2] |
| Number of adults per home, mean (SD) | 1.0  [0.9,1.1] | 0.9  [0.8,1.1] | 0.9  [0.7,1.2] |
| Number of children <18y in the household, mean (SD) | 0.9  [0.8,1.0] | 0.9  [0.7,1.1] | 0.9  [0.7,1.1] |
| Access to electronic devices, [Ref: None] |  |  |  |
| 1 to 2 | 0.3*  [0.1,0.9] | 0.2  [0.0,3.4] | 1.0  [0.4,3.0] |
| 3 or more | 0.3*  [0.1,0.8] | 0.2  [0.0,2.2] | 0.9  [0.3,3.1] |
| Electronic device in the room | 1.0  [0.8,1.1] | 1.4  [1.0,2.1] | 1.0  [0.6,1.6] |
| Limits in the use of electronic devices | 1.1  [0.9,1.3] | 0.9  [0.6,1.3] | 0.8  [0.5,1.3] |
| Available space to play | 1.1  [0.8,1.6] | 0.6  [0.3,1.2] | 0.7  [0.4,1.4] |
| Available backyard | 1.1  [0.8,1.6] | 1.3  [0.7,2.2] | 0.9  [0.5,1.6] |
| Income level (Ref: Low) |  |  |  |
| Medium | 1.3*  [1.0,1.6] | 2.0  [0.5,8.3] | 1.1  [0.6,2.0] |
| High | 2.0***  [1.4,2.7] | 2.9  [0.7,12.5] | 2.0  [0.3,15.1] |
| Rural area, (Ref: Urban) | 0.9  [0.7,1.1] | 0.6  [0.1,2.4] | 1.4  [0.9,2.3] |
| Confinement | 1.1  [0.9,1.4 | 1.0  [0.6,1.8] | 0.9  [0.5,1.6] |

OR, Odds Ratio; 95% CI, 95% Confidence Intervals

^a^Models adjusted for the variables listed in the table, meeting sleep time recommendation before COVID-19 and country region

**Supplementary eTable 4. Logistic regression of factors associated with having good sleep quality during COVID-19 across sample characteristics, by country.**

|  | **Chile**  **(n=3045)** | **Mexico**  **(n=632)** | **US Latinos**  **(n=459)** |
| --- | --- | --- | --- |
| **Associated factors** | **OR (95% CI) ^a^** | **OR (95% CI)^a^** | **OR (95% CI)^a^** |
| **Meeting other movement behavior recommendations** |  |  |  |
| Physical activity | 1.3**  [1.1,1.7] | 2.0**  [1.3,3.2] | 1.8*  [1.1,3.0] |
| Screen time | 1.1  [0.7,1.7] | 2.4  [0.8,6.9] | 1.4  [0.5,3.5] |
| **Child characteristics** |  |  |  |
| Male sex, (Ref: Female) | 1.0  [0.8,1.2] | 1.1  [0.8,1.7] | 0.8  [0.5,1.3] |
| Age, (Ref: 1-2 y) |  |  |  |
| 3-4 y | 1.519***  [1.204,1.917] | 2.2**  [1.3,3.7] | 1.2  [0.7,2.1] |
| 5 y | 1.4*  [1.2,1.9] | 1.4  [0.8,2.5] | 1.1  [0.543,2.257] |
| Enrolled in early childcare center | 1.0  [0.8,1.3] | 0.8  [0.5,1.3] | 0.9  [0.5,1.5] |
| Children usually plays with someone | 1.1  [0.8,1.3] | 1.1  [0.7,1.8] | 1.4  [0.8,2.3] |
| **Caregiver characteristics** |  |  |  |
| Age, (Ref: 30 years or less) |  |  |  |
| 31-40 years | 1.1  [0.9,1.4] | 0.9  [0.6,1.5] | 0.9  [0.5,1.5] |
| 41 years or more | 1.3  [0.8,2.0] | 0.6  [0.3,1.2] | 1.1  [0.5,2.8] |
| Male sex, (Ref: Female) | 1.8  [1.0,3.3] | 0.9  [0.4,1.9] | 2.5  [0.3,20.9] |
| Education level, (Ref: Incomplete high school or less) |  |  |  |
| Complete high school or technical degree | 1.1  [0.8,1.4] | 1.0  [0.3,3.0] | 1.0  [0.6,1.8] |
| University degree | 0.9  [0.6,1.3] | 1.6  [0.5,4.7] | 1.1  [0.5,2.1] |
| **Household characteristics** |  |  |  |
| Housing type, (Ref: House) |  |  |  |
| Apartment or Condominium | 1.0  [0.8,1.4] | 1.4  [0.8,2.6] | 1.4  [0.8,2.5] |
| Other | 0.9  [0.5,1.5] | 1.4  [0.1,27.7] | 1.2  [0.5,2.6] |
| Number of adults per home, mean (SD) | 1.0  [0.9,1.1] | 1.0  [0.7,1.3] | 1.1  [0.9,1.5] |
| Number of children <18y in the household, mean (SD) | 0.9  [0.8,1.1] | 1.1  [0.8,1.4] | 0.9  [0.7,1.2] |
| Access to electronic devices, [Ref: None] |  |  |  |
| 1 to 2 | 1.0  [0.4,2.6] | 4.4*  [1.4,14.5] | 0.9  [0.2,3.5] |
| 3 or more | 1.0  [0.4,2.8] | 3.7*  [1.1,12.5] | 0.9  [0.2,4.2] |
| Electronic device in the room | 0.8*  [0.7,1.0] | 0.9  [0.6,1.4] | 0.6*  [0.4,1.0] |
| Limits in the use of electronic devices | 1.0  [0.8,1.2] | 0.9  [0.6,1.4] | 1.1  [0.6,1.8] |
| Available space to play | 1.9***  [1.3,2.7] | 2.2*  [1.1,4.5] | 1.4  [0.7,2.8] |
| Available backyard | 1.0  [0.7,1.5] | 0.9  [0.5,1.7] | 1.2  [0.6,2.2] |
| Income level (Ref: Low) |  |  |  |
| Medium | 1.1  [0.9,1.4] | 0.7  [0.1,3.3] | 1.5  [0.8,3.0] |
| High | 1.5**  [1.1,2.1] | 1.0  [0.2,5.2] | 4.9  [0.4,64.4] |
| Rural area, (Ref: Urban) | 1.2  [0.9,1.6] | 3.4  [0.3,35.1] | 0.9  [0.6,1.6] |
| Confinement | 0.8  [0.7,1.0] | 0.9  [0.5,1.6] | 0.9  [0.5,1.8] |

OR, Odds Ratio; 95% CI, 95% Confidence Intervals

^a^Models adjusted for the variables listed in the table, meeting screen time recommendation before COVID-19 and country region
